# The Prevalence of Drug-Resistant Tuberculosis in Mainland China: An Updated Systematic Review and Meta-Analysis

**DOI:** 10.1371/journal.pone.0148041

**Published:** 2016-02-09

**Authors:** Qionghong Duan, Zi Chen, Cong Chen, Zhengbin Zhang, Zhouqin Lu, Yalong Yang, Lin Zhang

**Affiliations:** 1 Department of Tuberculosis Prevention, Wuhan Tuberculosis Institution, Wuhan, Hubei, China; 2 Department of Thyroid-Breast Surgery, Tongji Hospital, Tongji Medical College, Huazhong University of Science and Technology, Wuhan, Hubei, China; The Foundation for Medical Research, INDIA

## Abstract

**Background:**

In recent years, drug resistant tuberculosis (DR-TB) particularly the emergence of multi-drug-resistant tuberculosis (MDR-TB) has become a major public health issue. The most recent study regarding the prevalence of drug-resistant tuberculosis in mainland China was a meta-analysis published in 2011, and the subjects from the included studies were mostly enrolled before 2008, thus making it now obsolete. Current data on the national prevalence of DR-TB is needed. This review aims to provide a comprehensive and up-to-date assessment of the status of DR-TB epidemic in mainland China.

**Methods:**

A systematic review and meta-analysis of studies regarding the prevalence of drug-resistant tuberculosis in mainland China was performed. Pubmed/MEDLINE, EMBASE, the Cochrane central database, the Chinese Biomedical Literature Database and the China National Knowledge Infrastructure Database were searched for studies relevant to drug-resistant tuberculosis that were published between January 1, 2012 and May 18, 2015. Comprehensive Meta-Analysis (V2.2, Biostat) software was used to analyse the data.

**Results:**

A total of fifty-nine articles, published from 2012 to 2015, were included in our review. The result of this meta-analysis demonstrated that among new cases, the rate of resistance to any drug was 20.1% (18.0%–22.3%; n/N = 7203/34314) and among retreatment cases, the rate was 49.8% (46.0%–53.6%; n/N = 4155/8291). Multi-drug resistance among new and retreatment cases was 4.8% (4.0%–5.7%; n/N = 2300/42946) and 26.3% (23.1%–29.7%; n/N = 3125/11589) respectively. The results were significantly heterogeneous (p<0.001, I^2^ tests). Resistance to isoniazid was the most common resistance observed, and HRSE (H: isoniazid; R: rifampicin; S: streptomycin; E: ethambutol) was the most common form for MDR among both new and retreatment cases. Different drug resistance patterns were found by subgroup analysis according to geographic areas, subject enrolment time, and methods of drug susceptibility test (DST).

**Conclusions:**

The prevalence of resistance to any drug evidently dropped for both new and retreatment cases, and multi-drug resistance declined among new cases but became more prevalent among retreatment cases compared to the data before 2008. Therefore, drug-resistant tuberculosis, particularly multi-drug-resistant tuberculosis among retreatment TB cases is a public health issue in China that requires a constant attention in order to prevent increase in MDR-TB cases.

## Introduction

In recent years, drug resistant tuberculosis has been a major public health issue, particularly the emergence of MDR tuberculosis (TB). MDR is defined as the in vitro resistance to both rifampicin and isoniazid with or without resistance to other TB drugs and serves as a major challenge for TB control that may undermine recent achievements. WHO reported that there were an estimated 480,000 (range: 350,000‒610,000) new cases of MDR-TB worldwide, and approximately 210,000 (range: 130,000–290,000) deaths from MDR-TB in 2013[1]. China is among the 27 countries with a high MDR-TB burden, approximately 54,000 (range: 48,000–61,000) cases of MDR-TB emerged among notified pulmonary TB cases in 2013, which made China second only to India[1].

There were two **National Tuberculosis Epidemiological Surveys** (the fourth in 2000 and the fifth in 2010) that included data regarding the prevalence of DR-TB nation-wide. In the fourth national survey, the prevalence of any drug resistance (resistance to at least one of the four first-line anti-TB drugs) in pulmonary TBs was 27.8% (MDR was 10.7%)[2]. In the fifth national survey, the prevalence of any drug resistance was 36.8% (MDR was 6.8%)[3]. In 2007–2008, the **National Baseline Survey of Drug-resistant Tuberculosis** was conducted, which showed that the prevalence of any drug resistance among new smear positive pulmonary TB cases was 33.9% (mono-drug was 19.9% and multi-drug was 5.7% for the four first-line drugs) and among retreatment cases was 53.3% (mono-drug was 18.9% and multi-drug was 25.6% for the four first-line drugs)[4].

In the past decade, studies on the prevalence of DR-TB in China have been published, but the data were collected among different settings and were inconsistent. The most recent study regarding the prevalence of DR-TB in mainland China was a meta-analysis published in 2011[5],in which the subjects from the included studies were mostly enrolled before 2008, thus making it now obsolete. More current data regarding the national prevalence of DR-TB is needed. Therefore, this review aims to provide a comprehensive and up-to-date assessment of the status of the DR-TB epidemic in mainland China.

## Materials and Methods

### Literature Identification

Literature search methods included scouring original articles in the following electronic databases: Pubmed/MEDLINE, EMBASE, the Cochrane central database, the Chinese BioMedical Literature Database and the China National Knowledge Infrastructure Database from January 1, 2012 to May 18, 2015. Literature languages were restricted to English and Chinese. The keyword and medical subject heading search were combined as the search strategy. Search terms included “tuberculosis” or “Mycobacterium tuberculosis” and “drug resistance” or “drug susceptibility” and “China”.

### Inclusion and Exclusion criteria

Any article that reported the prevalence of any drug, mono-drug or multi-drug resistance was included. Only studies containing data regarding the prevalence of DR-TB among new and retreatment cases or both were accepted. Appraisal tool, developed in the Agency for Healthcare Research and Quality (AHRQ) for judging methodological quality of cross-sectional studies, was used to judge methodological quality[6]. It contains 11 items and each item is corresponding to a “Yes”, “No”, or “Unclear”. Since item 5, 7, 8, 9 and 11 in the checklist were not correlated with the contents of our study, we only included the items of 1, 2, 3, 4, 6 and 10 in the AHRQ methodology checklist to evaluate the quality of methodology. Among these included items, the more “yes” answers given to the items, the better quality of the included studies. The criterion of exclusion was that less than four items were rated “yes”. In the case of repeated publication, only the first published article or the article in English was included. Article from Chinese non-scientific-key periodicals, reviews, studies based on same population, studies containing data before 2008, studies with incorrect data, studies without required data, studies not reporting the DST method, studies not reporting the sampling method, studies using a non-standard definition of drug resistance, and studies using a non-standard definition of new or retreatment cases were excluded.

### Data Extraction

Data extraction from the included articles was independently performed by two reviewers using a structured data extraction form. The difference between the two reviewers was investigated by the whole study team to achieve a final agreement. The following information was extracted from every included original article: the name of first author; the year of publication; the age and gender distribution of the study subject; the setting of the study; the methods of DST; and the prevalence of any, mono or multiple drug resistance according to the four first-line anti-TB drugs. The three DST methods used were the proportion method, the absolute concentration method and the BACTEC method, which used BACTEC 960 TB system to diagnose tuberculosis. The time period of subject enrolment was classified into between 2008 and 2011, between 2011 and 2014, and between 2008 and 2014.

In this article, “new cases” are defined as pulmonary TB cases that had never taken anti-TB drugs or had taken anti-TB drugs for less than one month. “Retreatment cases” are defined as pulmonary TB cases that had taken a course of anti-TB drugs for longer than one month. “Mono-drug resistance” refers to the resistance to only one of the four first-line anti-TB drug (H, R, S, E). ‘‘Multi-drug resistance” (MDR) refers to the resistance to at least isoniazid and rifampin. “Any drug resistance” refers to resistance to any of the four first-line anti-TB drug (H, R, S, E).

### Statistical analysis

Comprehensive Meta-Analysis (V2.2, Biostat) was used to conduct the meta-analysis[7]. The pooled prevalence of “Mono-drug resistance”, ‘‘Multi-drug resistance” and “Any drug resistance” among new or retreatment TB cases was calculated. A stratified analysis was conducted in accordance with the geographic areas, subject enrolment time and DST method (proportion method, absolute concentration method). Fixed effect or random effect models were selected based on the heterogeneity shown by the I^2^ test. Since the distribution of values in all of the meta-analyses exhibited significant heterogeneity, we used a random effect model for all meta-analyses. Publication bias was assessed by funnel plot, Egger’s weighted regression and Begg’s rank correlation methods. Rate differences among the subgroups were tested by chi-square tests. If a new cases group in an included study contained fewer than 100 cases or a retreatment group contained fewer than 20 cases, then the data for this group was excluded. After exclusion, a sensitivity analysis was performed to evaluate the effect of sample size on the pooled prevalence.

## Results

**Fig 1:**
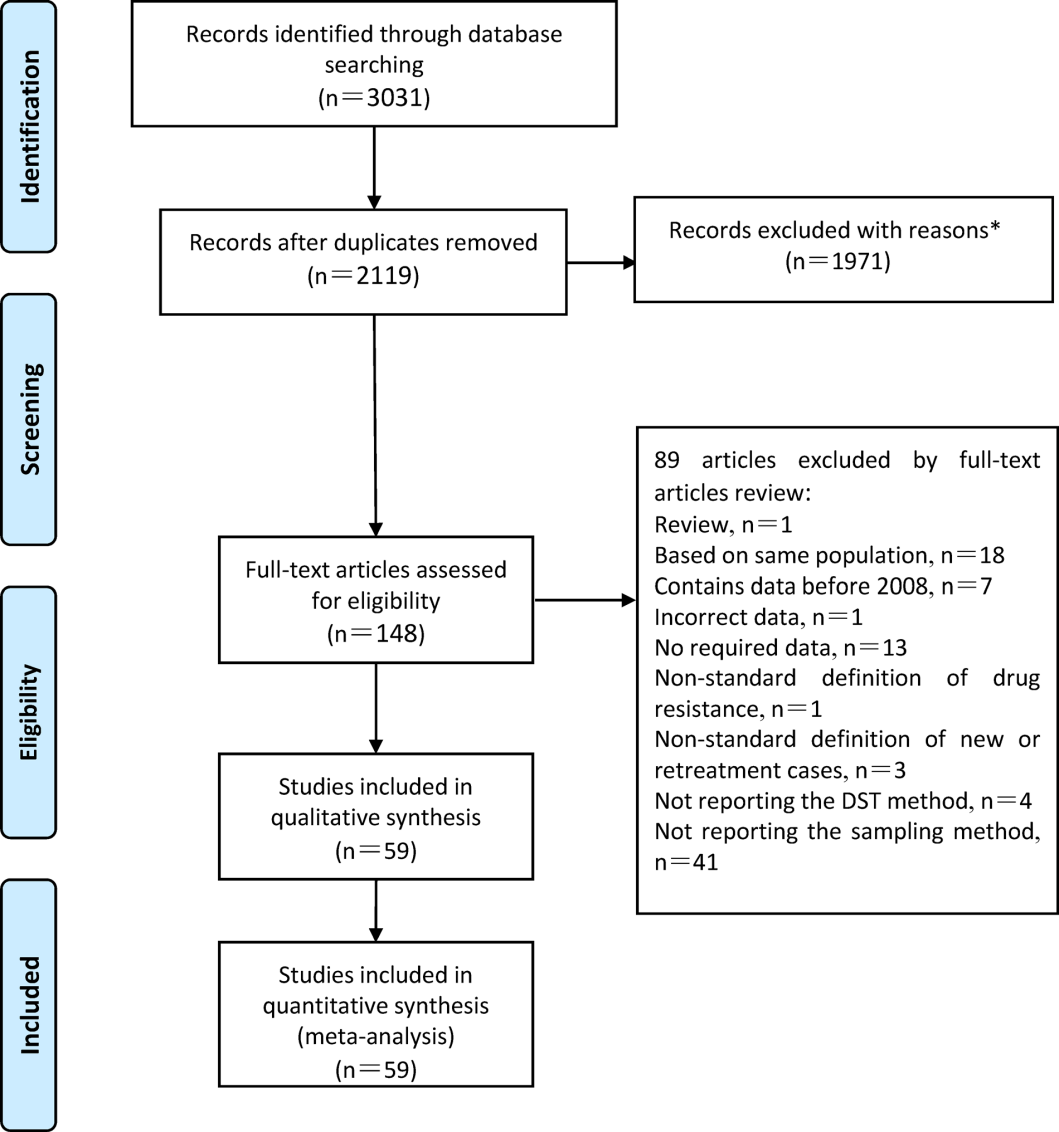
Flow chart depicting the study selection process. *The reasons including irrelevant topic, articles from Chinese non-scientific-key journals, review, insufficient data.

Three thousand and thirty-one articles were included by literature search, as shown in Fig 1. Two thousand one hundred and nineteen articles were retrieved after duplicates were removed. After an exclusion was performed based on an evaluation of titles and abstracts, 148 articles remained. Finally, through a detailed full-text evaluation, 59 articles were included. In our results, the number of the answers of included items in the AHRQ methodology checklist rated as “yes” was between 4 and 6. Therefore, the quality of included studies was good (**S1 Table**). **S2 Table** displays the included articles after the full-text evaluation.

As shown in **S3 Table**, of the 59 articles, 56 provided data on new cases and 55 provided data on retreatment cases. Regarding DST methods, 51, 7 and 1 studies used the proportion method, the absolute concentration method and the BACTEC, respectively. Based on the setting of study, 36, 17 and 8 studies were conducted in eastern, western, and central China. According to the subject enrolment time, 23, 13 and 24 studies were between 2008 and 2011, between 2011 and 2014 or between 2008 and 2014. Twenty-nine studies were conducted in urban areas, 3 studies in rural areas, and 27 in both urban and rural areas.

As displayed in **Table 1**, our meta-analysis produced result on the prevalence of drug-resistant TB among new cases of TB in China. Fifty-six studies were involved. The pooled prevalence of any drug resistance, mono-drug resistance and MDR were 20.1% (18.0%–22.3%; n/N = 7203/34314), 10.8% (9.3%–12.6%; n/N = 2761/23182) and 4.8% (4.0%–5.7%; n/N = 2300/42946), respectively. Significant heterogeneity was observed by the I^2^ test (p<0.001). **S1 Fig** shows a forest plot of the any drug resistance for new TB cases. As listed in **S2 Fig**, no significant publication bias was found (p = 0.9916 by Begg’s rank correlation analysis; p = 0.1221 by Egger’s weighted regression analysis). Using a stratified analysis, subject enrolment time (p<0.001) and DST methods (p<0.001) were found to significantly influence the prevalence of any drug resistance. The geographic areas did not significantly affect the prevalence (p = 0.460).

**Table 1:** Pooled prevalence of drug resistant tuberculosis among new cases in China.

		Prevalence of Drug Resistance (95% CI)	n/N	No. of Studies	Heterogeneity Test	
		(%)			I^2^ (%)	P
**Any drug resistance**	**Total**	20.1(18.0–22.3)	7203/34314	42	95.52	<0.001
	**Stratified by geographic areas**					
	Eastern China	20.7(18.1–23.5)	4658/22318	26	95.52	<0.001
	Central China	19.4(12.2–29.5)	678/3104	5	96.54	<0.001
	Western China	19.0(14.6–24.4)	1867/8892	11	95.83	<0.001
	**Stratified by years**					
	2008–2011	20.3(16.9–24.2)	2137/10163	15	93.16	<0.001
	2011–2014	19.1(15.8–23.0)	1953/10229	12	94.69	<0.001
	2008–2014	20.6(16.9–25.0)	3113/13922	16	96.89	<0.001
	**Stratified by DST methods**					
	Absolute concentration method	22.7(14.8–33.2)	1389/5330	4	96.45	<0.001
	The proportion method	19.8(17.7–22.1)	5814/28984	38	95.06	<0.001
**Mono-drug resistance**	**Total**	10.8(9.3–12.6)	2761/23182	37	93.56	<0.001
	**Stratified by geographic areas**					
	Eastern China	11.7(9.6–14.2)	1461/11418	21	93.27	<0.001
	Central China	12.5(5.5–25.9)	357/2635	4	97.49	<0.001
	Western China	9.0(7.1–11.3)	943/9129	12	87.28	<0.001
	**Stratified by years**					
	2008–2011	10.7(7.2–15.8)	403/3188	11	91.94	<0.001
	2011–2014	10.6(8.2–13.6)	522/4818	8	88.62	<0.001
	2008–2014	10.9(8.7–13.5)	1836/15176	18	95.28	<0.001
	**Stratified by DST methods**					
	Absolute concentration method	11.7(6.8–19.3)	639/5018	3	94.22	<0.001
	The proportion method	10.7(9.0–12.7)	2122/18164	34	93.70	<0.001
**Multi-drug resistance**	**Total**	4.8(4.0–5.7)	2300/42946	56	93.38	<0.001
	**Stratified by geographic areas**					
	Eastern China	4.7(3.8–5.8)	1229/26921	33	92.32	<0.001
	Central China	4.2(2.7–6.5)	208/4282	8	85.93	<0.001
	Western China	5.4(3.8–7.5)	863/11743	17	94.09	<0.001
	**Stratified by years**					
	2008–2011	4.2(3.2–5.5)	495/12302	21	83.47	<0.001
	2011–2014	4.6(3.3–6.5)	475/10963	13	92.81	<0.001
	2008–2014	5.6(4.3–7.2)	1330/19681	23	95.14	<0.001
	**Stratified by DST methods**					
	Absolute concentration method	5.0(3.5–7.1)	420/6232	6	82.04	<0.001
	The proportion method	4.7(3.8–5.7)	1791/36006	49	93.58	<0.001

**Table 2** shows the result of the meta-analysis on the prevalence of drug resistant TB among retreatment cases in China. Fifty-fives tudies were involved. The pooled prevalence of any drug resistance, mono-drug resistance and MDR was 49.8% (46.0%–53.6%; n/N = 4155/8291), 12.8% (11.0%–14.9%; n/N = 857/6252) and 26.3% (23.1%–29.7%; n/N = 3125/11589), respectively. Significant heterogeneity was observed by I^2^ test (p<0.001). **S3 Fig** shows a forest plot of the any drug resistance for retreatment cases. As listed in **S4 Fig**, no significant publication bias was found (p = 0.8753 by Begg’s rank correlation analysis; p = 0.9065 by Egger’s weighted regression analysis). Using a stratified analysis, geographic areas (p = 0.012), DST methods (p<0.001), and the subject enrolment time (p<0.001) were considered to significantly influence the prevalence of any drug resistance.

**Table 2:** Pooled prevalence of drug resistant tuberculosis among retreatment cases in China.

		Prevalence of Drug Resistance (95% CI)	n/N	No. of Studies	Heterogeneity Test	
		(%)			I^2^ (%)	P
**Any drug resistance**	**Total**	49.8(46.0–53.6)	4155/8291	42	90.39	<0.001
	**Stratified by geographic areas**					
	Eastern China	51.9(47.2–56.5)	2511/4891	26	89.76	<0.001
	Central China	45.1(37.2–53.2)	697/1480	5	83.30	<0.001
	Western China	46.5(36.8–56.4)	947/1920	11	93.39	<0.001
	**Stratified by years**					
	2008–2011	48.8(44.5–53.1)	1188/2460	16	68.86	<0.001
	2011–2014	47.8(41.6–54.1)	1146/2417	11	87.84	<0.001
	2008–2014	51.7(43.9–59.5)	1821/3414	16	94.75	<0.001
	**Stratified by DST methods**					
	Absolute concentration method	57.8(44.6–69.9)	565/872	5	90.69	<0.001
	The proportion method	48.7(45.0–52.4)	3590/7419	37	88.41	<0.001
**Mono-drug resistance**	**Total**	12.8(11.0–14.9)	857/6252	37	78.38	<0.001
	**Stratified by geographic areas**					
	Eastern China	12.4(10.3–15.0)	428/3339	21	73.69	<0.001
	Central China	17.2(11.2–25.4)	174/945	4	76.44	0.005
	Western China	12.1(8.7–16.7)	255/1968	12	82.34	<0.001
	**Stratified by years**					
	2008–2011	14.6(11.2–18.8)	148/1043	12	55.44	<0.01
	2011–2014	13.1(9.3–18.2)	250/1645	7	81.96	<0.001
	2008–2014	11.8(9.3–14.9)	459/3564	18	83.71	<0.001
	**Stratified by DST methods**					
	Absolute concentration method	8.8(5.3–14.2)	80/832	4	72.17	0.013
	The proportion method	13.4(11.5–15.7)	777/5420	33	77.39	<0.001
**Multi-drug resistance**	**Total**	26.3(23.1–29.7)	3125/11589	55	92.90	<0.001
	**Stratified by geographic areas**					
	Eastern China	29.9(26.4–33.6)	1738/5786	33	87.43	<0.001
	Central China	17.7(13.7–22.6)	293/1617	7	70.79	0.002
	Western China	22.9(16.3–31.1)	1094/4186	17	96.15	<0.001
	**Stratified by years**					
	2008–2011	23.9(20.9–27.3)	668/2890	21	63.56	<0.001
	2011–2014	25.9(20.1–32.7)	635/2632	12	91.62	<0.001
	2008–2014	28.8(23.1–35.3)	1822/6067	23	95.92	<0.001
	**Stratified by DST methods**					
	Absolute concentration method	29.9(20.8–41.0)	425/1050	7	90.53	<0.001
	The proportion method	26.2(23.1–29.6)	2540/9354	47	90.88	<0.001

In **S4 Table**, the meta-analysis of the distribution of drug resistance patterns in new casesis listed. Isoniazid and streptomycin were found to be the most common drug resistance, with a pooled any drug prevalence of 12.0% (10.6%–13.6%) and 11.8% (10.1%–13.7%), respectively. The most common type of drug resistance was HRSE, with a prevalence of 1.4% (1.1%–1.9%) among MDR-TB cases. **S4 Table** also shows the meta-analysis of anti-TB drug resistance patterns in retreatment cases. Similarly, isoniazid and rifampicin were founded to be the most common resistance with a pooled prevalence of 40.0% (35.8%–44.3%) and 33.3% (29.6%–37.2%), respectively. The most common form of drug resistance was HRSE, with a prevalence of 8.5% (6.5%–11.1%) among MDR-TB.

According to the results of the sensitivity analysis, the prevalence of any drug resistance was not significantly verified in new cases as 19.8% (17.8%–22.1%; p = 0.9552), excluding the data of new cases with a sample size smaller than 100. The prevalence of any drug resistance was not significantly verified in retreatment cases as 50.0% (46.1%–53.8%; p = 0.9686), excluding the data of retreatment cases with a sample size smaller than 20.

## Discussion

This is an updated review of drug-resistant TB in mainland China. In this meta-analysis, 59 studies were included. The pooled prevalence of drug-resistant TB among new cases was 20.1% (18.0%-22.3%), and among retreatment cases, it was 49.8% (46.0%-53.6%). The pooled prevalence of mono-drug-resistant TB among new cases was 10.8% (9.3%-12.6%), and among retreatment cases, it was 12.8% (11.0%-14.9%). The pooled prevalence of MDR-TB among new cases was 4.8% (4.0%-5.7%), and among retreatment cases, it was 26.3% (23.1%-29.7%). Different drug resistant patterns were presented among different subgroups categorized by geographic areas, subject enrolment time and DST methods.

Ever since the discovery of anti-TB drugs, resistance to these agents has been ever-present due to irresponsible abuse of these precious antibiotics. MDR-TB, a TB strain that is resistant to at least two of the most important anti-TB drugs (isoniazid and rifampicin), is more difficult to diagnose and treat than drug-susceptible TB[8]. Furthermore, the development of new anti-TB drugs lag behind the alarming spread of drug resistance[9,10]. Consequently, DR-TB (especially MDR-TB) has posed a challenge to the overall control of TB. In China, the problem of DR-TB has drawn increasing attention. In 2007–2008, the **National Baseline Survey of Drug-Resistant Tuberculosis** was successfully conducted. In the **National tuberculosis prevention and control guideline** (2008 version), the detection and management of DR-TB cases was highlighted. The **Guideline for the Treatment of Drug-Resistant Tuberculosis** (2009), published by the Chinese Anti-Tuberculosis Association, was composed to further standardise the MDR-TB chemotherapy and to reduce the incidence of MDR-TB.

The typical acquired resistance defined as DR-TB initially results from several anthropogenic factors, such as poor-quality anti-TB drugs, poor treatment adherence and inadequate or improper treatment[11–13]. An initial drug resistance is when a direct infection of drug-resistant strain occurs. Once the drug resistance occurs, DR-TB cases will be turned into a source of infection, transmitting the disease directly to other people. Although it is not definitive to classify initial and acquired drug resistance by the history of treatment[14–18], the initial drug resistance is normally found among newly diagnosed TB cases. In our meta-analysis, 20.1% of new TB cases were resistant to at least one anti-TB drug, and 4.8% of new cases were MDR-TB. The initial MDR-TB infection was higher than the global average (3.5%) but was in the range of the WHO's estimation for China (5.7%, 4.5%-7.0%). Therefore, the early detection and effective treatment of the source of infection is crucial in terms of reducing the DR-TB transmission. Acquired drug resistance is usually found among retreatment TB cases. In our meta-analysis, 49.8% of retreatment TB cases were resistant to at least one anti-TB drug, and 26.3% of retreatment cases were MDR-TB. Acquired MDR-TB infection was higher than the global average (20.5%), but was very close to WHO's estimation for China (26.0%, 22.0%-30.0%). Thus, we should place an emphasis on the management and treatment of patients.

A stratified analysis was conducted to study the characteristics shown in the subgroups categorized by geographic areas, subject enrolment time, and DST methods. The greatest number of studies was from eastern China, followed by studies from western regions, with the remainder of the studies from central China. The eastern China had significantly higher any drug resistance prevalence among new and retreatment cases than the rest of China. The highest MDR-TB prevalence among retreatment cases was also found in that region. In the **National Baseline Survey of Drug-resistant Tuberculosis** (2007–2008), the difference in the any drug resistance prevalence among the different regions was not significant, where as the prevalence of MDR among retreatment TB cases in eastern China was significantly higher than that of western China. These results revealed an expanding trend in differences in the prevalence of DR-TB between the eastern and western region. Although eastern China enjoys a higher social economic status, which leads to a smaller TB epidemic, eastern China is worse in terms of drug-resistance situation. We should therefore pay closer attention on controlling tuberculosis drug resistance in eastern China. We also found that the central China had the lowest MDR-TB prevalence among new and retreatment cases. In the **National Baseline Survey of Drug-resistant Tuberculosis** (2007–2008), the central China had a modest MDR-TB prevalence among new cases, and the highest MDR-TB prevalence among retreatment cases. Thus, the condition of MDR-TB in the central China has been improved.

There are 3 different DST methods: the absolute concentration method, the proportion method, and the BACTEC method. There was only 1 study using the BACTEC method; therefore, the BACTEC method was not included in the DST methods subgroup. The prevalence of drug resistance was lower in the proportion method group than in the absolute concentration method group. Therefore, when the results of different studies are compared, different DST methods should be considered.

Comparing the results of this study to those of the **National Baseline Survey of Drug-Resistant Tuberculosis** (2007–2008), the prevalence of any drug-resistant TB evidently decreased for both new and retreatment TB cases (20.1% vs. 33.9% for new cases, 49.8% vs. 53.3% for retreatment cases). Although an MDR-TB decline was observed among new cases (4.8% vs. 5.7%), there was an increased prevalence among retreatment cases (26.3% vs. 25.6%). When compared to the meta-analysis in 2011, the results of the current study revealed that the prevalence of any drug resistance among new cases (27.9% vs. 20.1%) and retreatment cases (60.3% vs. 49.8%) and MDR-TB among new cases (5.3% vs. 4.8%) showed clearly declining trends, where as MDR-TB among retreatment TB cases (27.4% vs. 26.3%) only slightly declined. In the **National Tuberculosis Epidemiological Survey**, the overall prevalence of drug resistance was much lower than the aforementioned results, which may in part be due to the different subject enrolment methods among the different surveys. The subjects in the **National Tuberculosis Epidemiological Survey** were screened from the general population, where as the subjects in the **National Baseline Survey of Drug-Resistant Tuberculosis** were registered TB cases. Thus, the proportion of relapsed and chronically relapsed TB cases in the **National Tuberculosis Epidemiological Survey** was lower than in the **National Baseline Survey of Drug-Resistant Tuberculosis**. As a result, such inconsistencies may influence the reported prevalence of drug resistance. In terms of subject enrolment time, an upward trend of MDR-TB prevalence among new and retreatment TB cases was observed, which indicates that the condition of multi-drug resistance has continued to deteriorate from 2011 to the present day. Therefore, DR-TB, particularly MDR-TB among retreatment TB cases is becoming a major characteristic of DR-TB epidemic, which requires specific attention and effective measures to address it.

This study had several limitations. First, the majority of the studies were conducted in urban areas or both urban and rural areas, with only a few studies from rural areas. The subjects in most studies were enrolled in hospitals, whereas others were screened from the general population. Those aforementioned two factors may lead to selection bias. Second, potential publication bias and language bias could not be completely excluded. Third, significant heterogeneity was observed among certain studies. There were great degrees of inconsistency among demographic information of subjects, standards of quality, and methodologies, which were adopted by different studies. Those inconsistencies would certainly influence the results of this meta-analysis. Although we conducted a subgroup analysis to explain this heterogeneity, the information from the subgroup analysis was insufficient because included studies had a limited amount of the aforementioned information. Consequently, there remains great confusion concerning this heterogeneity.

In conclusion, this is an updated systematic review and meta-analysis on the prevalence of drug-resistant tuberculosis in mainland China. This study attempts to demonstrate the drug-resistance status in mainland China after 2008. The any drug resistance prevalence dropped significantly among both new and retreatment TB cases, where as MDR-TB evidently dropped among new cases but became more prevalent among retreatment cases compared with the pre-2008 data. These findings highlight an urgent need for immediate measures to be taken to control DR-TB.

## Supporting Information

S1 Fig Forest plot (any drug resistance in new cases). (‘‘*”indicates studies reported by the same author but different publications).
(TIFF)

S2 Fig Funnel plot (any drug resistance in new cases). (TIFF)

S3 Fig Forest plot (any drug resistance in retreatment cases). (‘‘*”indicates studies reported by the same author but different publications).
(TIFF)

S4 Fig Funnel plot (any drug resistance in retreatment cases). (TIFF)

S1 Table Quality assessment checklist for included articles. (XLS)

S2 Table Included articles after full-text evaluation. (DOCX)

S3 Table Detailed data of the included studies. (XLS)

S4 Table Distribution of different drug resistance patterns among new and retreatment cases in China. (DOCX)

S5 Table PRISMA 2009 checklist. (DOC)
