# Supplementary figures and images for: The Prevalence of Drug-Resistant Tuberculosis in Mainland China: An Updated Systematic Review and Meta-Analysis
Source: PLoS One. 2016 Feb 9;11(2):e0148041. doi: 10.1371/journal.pone.0148041 (PMC4747587; doi:10.1371/journal.pone.0148041)

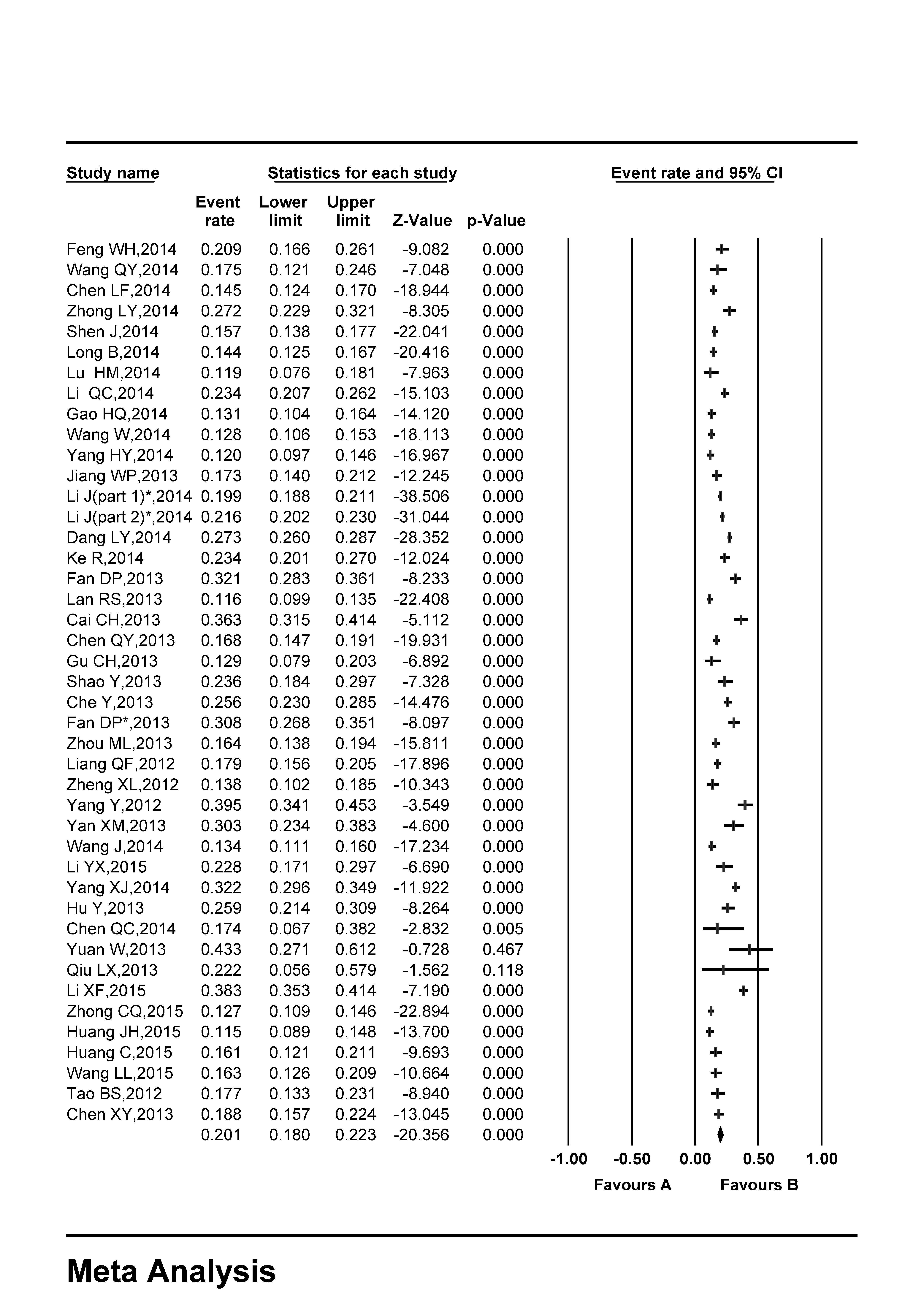

Supplement: S1 Fig — (‘‘*”indicates studies reported by the same author but different publications). (TIFF) [file pone.0148041.s001.tiff]

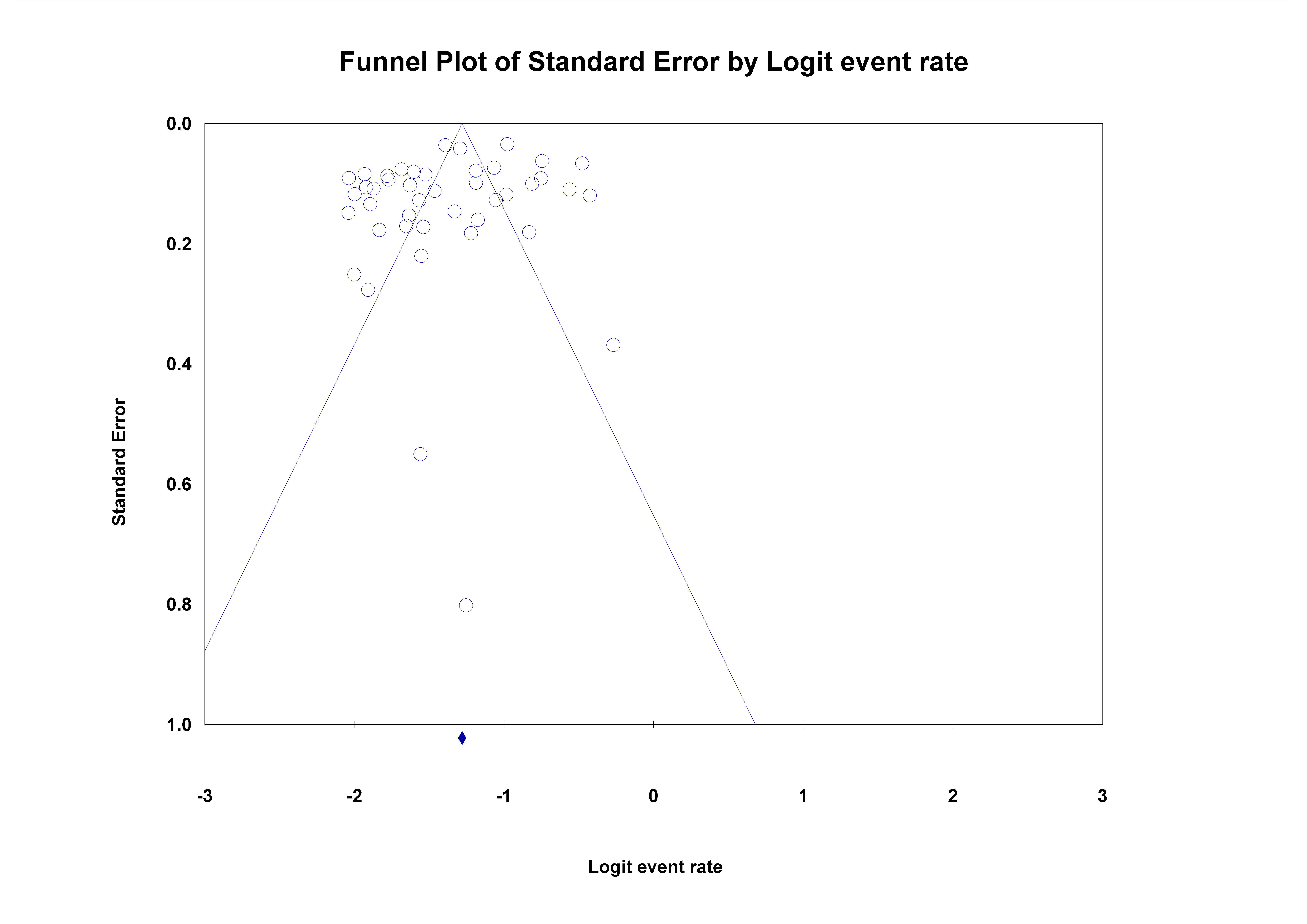

Supplement: S2 Fig — (TIFF) [file pone.0148041.s002.tiff]

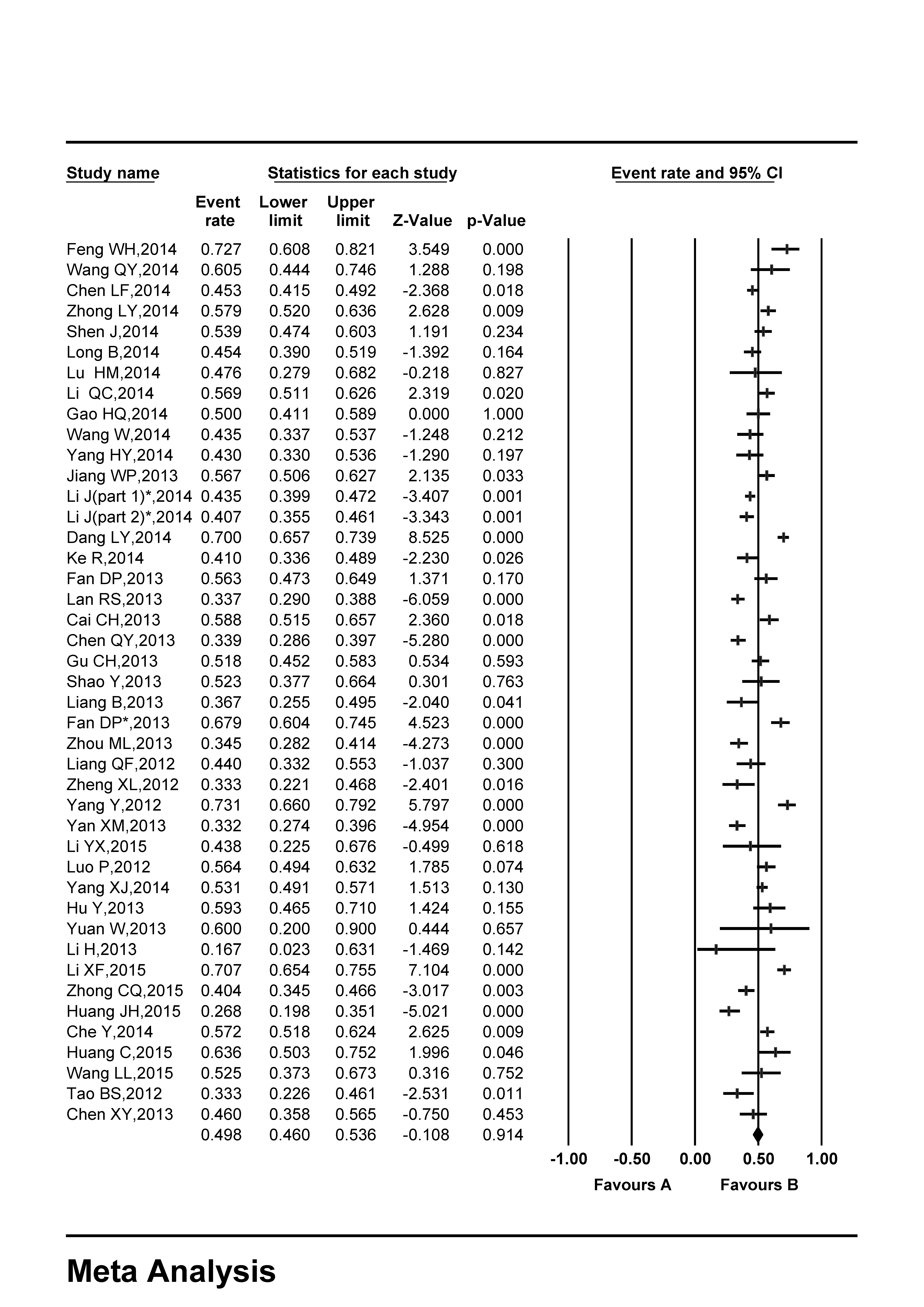

Supplement: S3 Fig — (‘‘*”indicates studies reported by the same author but different publications). (TIFF) [file pone.0148041.s003.tiff]

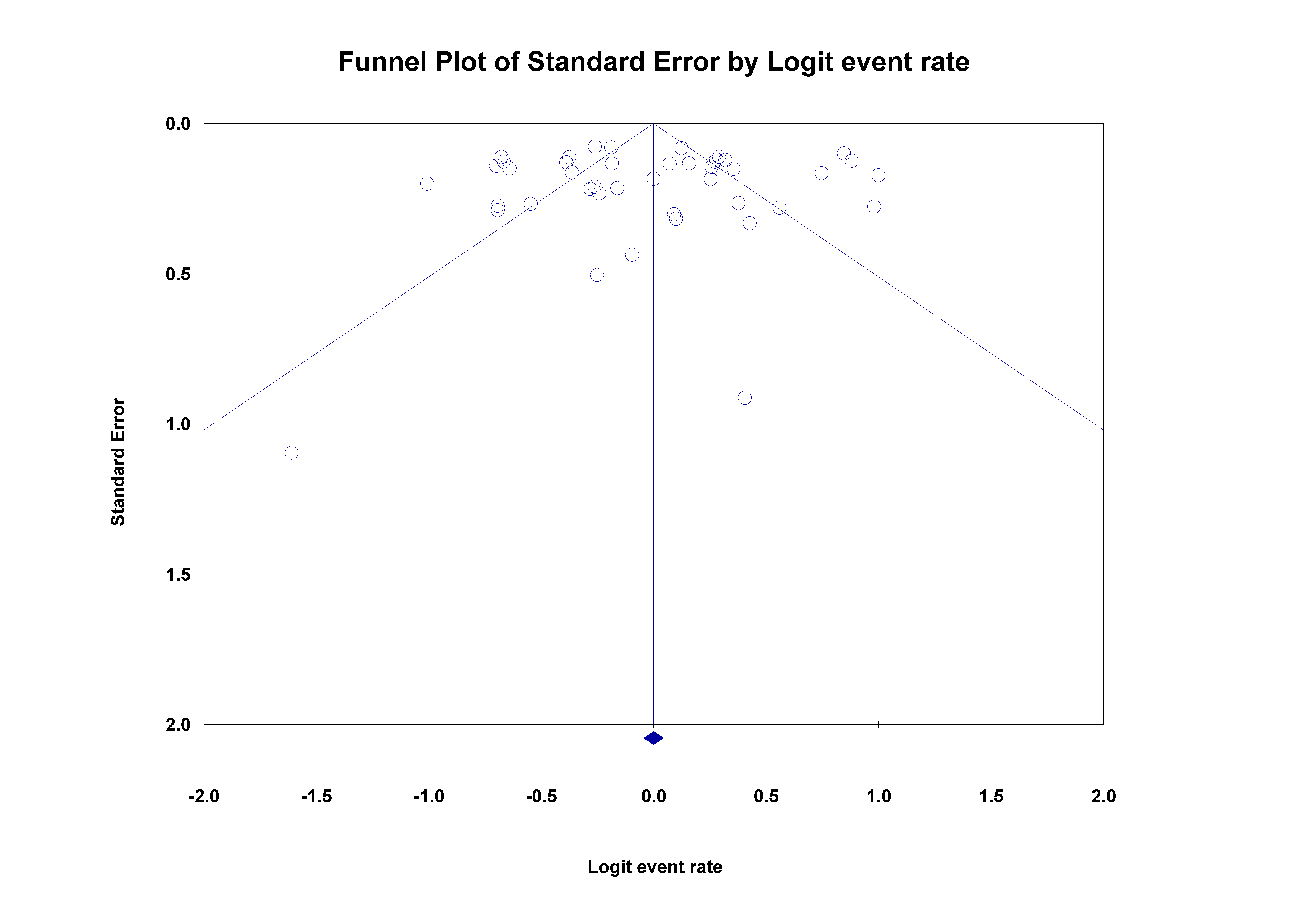

Supplement: S4 Fig — (TIFF) [file pone.0148041.s004.tiff]
