# Supplementary material for: The Prevalence of Drug-Resistant Tuberculosis in Mainland China: An Updated Systematic Review and Meta-Analysis
Source: PLoS One. 2016 Feb 9;11(2):e0148041. doi: 10.1371/journal.pone.0148041 (PMC4747587; doi:10.1371/journal.pone.0148041)
Supplement: S2 Table — (DOCX) [file pone.0148041.s006.docx]

## Table S2. Included articles after full-text evaluation

1. Luo LY,Bi ZY,Jiang DQ,Xu SA,Nie SQ (2014) Tuberculosis drug resistance and major epidemiological characteristics analysis of Langfang city. Journal of Medical Pest control 30:1188-1193.

2. Han YH (2014) Analysis of drug resistance in patients with pulmonary tuberculosis in Tangshan City. Hebei Medical Journal 36:1406-1408.

3. Feng WH (2014) Analysis of drug resistance in initial and retreated smear-positive tuberculosis patients in Handan city in 2013. China Tropical Medicine 14:1129-1130.

4. Wang QY,Hu DY,Liu Y,Shen J (2014) Study on the status of drug-resistant tuberculosis of urban area of Chongqing. Chongqing Medicine 43:2913-2915.

5. Chen LF,Zhou J,Chen J,Rao YY (2014) Analysis of drug resistance of 1561 tuberculosis patients in Wuhan. Journal of Clinical Pulmonary Medicine 19:1638-1641.

6. Zhong LY,li J,Xu AD,Huang GL (2014) New Monitoring Results of Tuberculosis Drug Resistance and Its Trend in Huizhou. Practical Journal of Cardiovascular and Cerebrovascular Diseases 22:36-38.

7. Shen J,Liu J,Chen L,Zhu DM,Wang XY,et al. (2014) Analysis of drug resistance on tuberculosis in six districts (counties) of Three Gorges Reservoir Area in 2010-2013. Chinese Journal of Antituberculosis 36:599-602.

8. Long B,Yang J,Wang WN,Gao WF,Chen Q,et al. (2014) Drug Resistance of Mycobacterium Tuberculosis in Sichuan. Journal of Preventive Medicine Information 30:430-433.

9. Lu HM,Zhang SC (2014) Analysis on the surveillance of TB drug resistance between 2009 and 2010 in Changxing county. Modern Preventive Medicine 41:1520-1521.

10. Ma BQ,Sun JT,Liu HJ,Zhu L,Li HW (2014) Analysis of drug resistance of 1119 Mycobacterium tuberculosis strains in Puyang. Chinese Journal of Antituberculosis 36:279-285.

11. Li QC, Wu LM,Lu M,Liu W,Jin HY,et al. (2014) Surveillance for tuberculosis drug resistance in Hangzhou, Zhejiang. Disease Surveillance 29:210-214.

12. Gao HQ,Chen QF,Jin FX, Niu WK (2014) Analysis on the status of drug -resistance of tuberculosis in Shaoxing City. Zhejiang Journal of Preventive Medicine 26:242-244.

13. Wang W,Hao XG (2013) Analysis of drug resistance status of mycobacterium tuberculosis in Quzhou from 2011 to 2012. Chinese Journal of Health Laboratory Technology 23:3601-3602,3613.

14. Yang HY,Zou YX,Shi YH (2014) A comparison study on the multi-drug resistance of tuberculosis in Huzhou City. Zhejiang Journal of Preventive Medicine 26:233-236.

15. Jiang WP,Sun CH (2013) Analysis of drug resistance of 680 strains of Mycobacterium tuberculosis. Journal of Public Health and Preventive Medicine 24:102-103.

16. Fang MT,Liu HM,Su YF,Liu GH,Zeng JF,et al. (2014) Analysis of drug resistance of 628 cases of pulmonary tuberculosis patients in hospital. Chinese Journal of Antituberculosis 36:131-133.

17. Li J,Zhang YY,Wu J,Jiang Y,Wang LL,et al. (2014) Trends of drug-resistant tuberculosis in Shanghai from 2007 to 2012. Chinese Journal of Antituberculosis 36:25-30.

18. Dang LY,Wei XL,Fang RT,Pang JJ,Zhang ZX,et al. (2014) Analysis of the drug-resistant status and risk factors of 4721 cases of hospitalized tuberculosis patients. Chinese Journal of Antituberculosis 36:49-54.

19. Ke R,Zheng RR,Zhang XD,Yang YM,Lin XL,et al. (2014) Analysis on epidemic and relevant factors for drug-resistant tuberculosis in Xiamen city. Chinese Journal of Antituberculosis 36:93-97.

20. Fan DP,Chen G,Wu SH,Yu DJ,Xu LH,et al. (2013) Analysis of Mycobacterium tuberculosis drug resistance in 723 tuberculosis patients in hospital. Zhejiang Journal of Preventive Medicine 25:42-48.

21. Lan RS,Lan L,Huang SH,Liu FY,Ou J,et al. (2013) Anti-tuberculosis drug resistance analysis of patients with smear positive pulmonary tuberculosis in Guangxi. Chinese Journal of Antituberculosis 35:673-678.

22. Cai CH,Zeng GY,Li LP,Huang Z (2013) Analysis on drug resistance of 540 strains of Mycobacterium tuberculosis. Zhejiang clinical medicine 15:1075-1076.

23. Chen QY,Lin SF,Liang QF,Wei SZ,Zhao Y,et al. (2013) Tuberculosis drug resistance surveillance report in Fujian province. Chinese Journal of Antituberculosis 35:511-515.

24. Lu W,Zhou Y,Chen C,Liu Q,Ding XY,et al. (2013) Prevalence and risk factors for drug resistance tuberculosis in Jiangsu Province:a population based study. Chinese Journal of Disease Control & Prevention 17:560-563.

25. Zhang JM,Jin WE,Yang RJ (2013) Surveillance of drug-resistance of Mycobacterium tuberculosis in Quzhou,Zhejiang. Disease Surveillance 28:322-325.

26. Gu CH,Wang Q,Wang FR,Wang XP (2013) Analysis of 340 tuberculosis patients with first-line anti-tuberculosis drug resistance monitoring results. Journal of Ningxia Medical College 35:74-76.

27. Shao Y,Song HH,Li GL,Yang DD,Liu Q,et al. (2013) Epidemiology of drug-resistant Mycobacterium tuberculosis strains circulating in Jiangsu Province in 2010. Journal of Nanjing Medical University (Natural Science) 33:282-285,290.

28. Che Y,Yu M,Ping GH,Lin X,Li ZB (2013) Analysis of the status and risk factors of drug resistant tuberculosis in Ningbo. Chinese Journal of Health Laboratory Technology 23:495-497.

29. Liang B,Wei YJ,Wu H,He QQ,Ouyang CH,et al. (2013) Characteristics and related factors of drug resistance in the retreatment smear positive pulmonary tuberculosis. Guangdong Medical Journal 34:277-279.

30. Shi ZY,Li RC,Duan BH,Yang YQ,Li CS,et al. (2012) Monitoring result analysis on resistance of 87 cases of tubercle bacillus in Da. Journal of Medical Pest Control 28:1315-1317.

31. Fan DP,Zhang Y,Xia Q,Yue YN,Zhu M (2012) Analysis of drug resistance of 636 pulmonary tuberculosis cases in Hangzhou city during 2010-2011. Chinese Journal of Antituherulosis 34:750-752.

32. Zhou ML,Fu ZJ,Wang JJ,Chen Z,Chen C,et al. (2012) Analysis on the results of the baseline survey of drug resistance of tuberculosis in Wuhan City. Journal of Public Health and Prevention Medicine 23:33-38.

33. Liang QF,Chen QY,Zhao Y,Wei SZ,Lin J,et al. (2012) Analysis of drug resistance surveillance of tuberculosis in Fujian province. Chinese Journal of Public Health 28:420-422.

34. Zheng XL,Wei JF,Wei BC,Pan DQ,Huang SH,et al. (2012) Investigation of smear positive pulmonary tuberculosis drug resistance status in the Guangxi Zhuang Autonomous Region Guigang City. Chinese Journal of Antituberculosis 34:404-406.

35. Mireban RXT,Hu X,Xu YL,Wang J,Muyeshaer BDY,et al. (2012) Analysis of drug resistances and strain types in 214 Mycobacterium tuberculosis isolates in Kashi region,Xinjiang. Chinese Journal of Antituberculosis 34:538-541.

36. Yang Y,Xie HB,Zheng CH,Weng JH,Chen ZJ (2012) Resistance of Mycobacterium tuberculosis to anti-TB drugs in diferent age group among inpatients with

pulmonary tuberculosis. China Tropical Medicine 12:64-66.

37. Deng JP,Li Q,Xiao TQ,Wang LL (2012) Preliminary analysis of the surveillance results on Mycobacterium tuberculosis drug resistance in Zigong City, Sichuan Province China. China Journal of Preventive Medicine 13:653-656.

38. Yan XM,Zhang Q,Zhang ZX (2013) Analysis of drug resistance of the 371 Mycobacterium tuberculosis strains from southern region of Xinjiang. Chinese Journal of Laboratory Diagnosis 17:688-691.

39. Wang J,Zhu M,Zhang Y,Yue YN,Hu YQ,et al. (2014) Analysis of drug resistance trend among newly registered TB patients Zhejiang Prevention Medicine 26:1240-1242.

40. Li YX,Pa ZR,Liu NQ. (2015) The drug resistance status of pulmonary tuberculosis patients in Bortala Mongolia Autonomous Prefecture in 2013. Chinese Journal of Antituberculosis 37:389-392.

41. Luo P,Zhang TH,Gao ZD,Xing Q,Zhao Y,et al. (2012) The study of drug resistance of retreatment tuberculosis patients in Beijing 2009-2010. Chinese Journal of Antituberculosis 34:704-707.

42. Yang XJ,Yuan Y,Pang Y,Wang B,Bai Y,et al. (2015) The burden of MDR/XDR tuberculosis in coastal plains population of China. PLoS One 10:e0117361.

43. Hu Y,Hoffner S,Wu L,Zhao Q,Jiang W,et al. (2013) Prevalence and genetic characterization of second-line drug-resistant and extensively drug-resistant Mycobacterium tuberculosis in Rural China. Antimicrob Agents Chemother 57:3857-3863.

44. Wang XM,Fu Q,Li Z,Chen S,Liu Z,et al. (2012) Drug-resistant tuberculosis in Zhejiang Province,China,1999-2008. Emerg Infect Dis 18:496-498.

45. Qi YC,Ma MJ,Li DJ,Chen MJ,Lu QB,et al. (2012) Multidrug-resistant and extensively drug-resistant tuberculosis in multi-ethnic region,Xinjiang Uygur Autonomous Region,China. PLoS One 7:e32103.

46. Yang XG,Yuan Y,Pang Y,Wang B,Bai Y,et al. (2012) The burden of MDR/XDR tuberculosis in coastal plains population of China. PLoS One 10:e0117361.

47. Chen QC,Tan SY,Xue ZQ,Wu GF,Lei Y,et al. (2014) Epidemiological survey of tuberculosis in Guangzhou city. Practical preventive medicine 21:437-440.

48. Yuan W,Zhang M,Chen YJ,Chen HJ,Li Y,et al. (2013) Bacteriological analysis and epidemiological survey of tuberculosis in five places of Guizhou province in 2010. Chinese Journal of Heath Laboratory Technology 23:1586-1589.

49. Li H,Mao XG,Shi J,Yan GR,Yang HY,et al. (2013) Analysis on bacteriology examination results of epidemiological sampling survey for tuberculosis sites of Henan province

in 2010. Chinese Journal of Antituberculosis 35:22-26.

50. Qiu LX,Shu Q (2013) Epidemiological sampling survey of tuberculosis in Jiangxi province,2010. Modern preventive medicine 40:1220-1224.

51. Li XF,Han Z,Ke XM (2015) Analysis on the screening results of drug-resistant tuberculosis in Huizhou city. Modern preventive medicine 22:527-530.

52. Cheng X,Li YW,Rao Y,Yang XH,Du H (2015) Drug resiatance sistuation in 2271 tuberculosis cases and epidemiological characteristics analysis. Chongqing Medicine 44:1635-1637.

53. Zhong CQ,Yang HS,Xu SZ,Fu X,Wang JM (2015) Surveillance of the drug resistance of tuberculosis in Lianyungang city during 2011-2013. Journal of medical research 44:45-48.

54. Huang JH,Shen XF,Zheng J,Bai JM,Liu J,et al. (2015) Characteristics and related factors of first-line anti-tuberculosis drug resistance among patients with pulmonary tuberculosis registered in Wuxi City. Modern preventive medicine 42:530-532.

55. Che Y,Yu M,Xu GZ,Ping GH (2014) Analysis on drug resistance of first-line anti-tuberculosis drugs in 332 pulmonary tuberculosis cases. Chinese Journal of health inspection 24:3481-3483.

56. Huang C,Wang XP,Gu XR,Ma JF,Lu F (2015) Prevalence of drug resistant tuberculosis in Haimen,Jiangsu. Disease surveillance 30:58-62.

57. Wang LL,Wu LP,Zhang JY,Ke WH (2012) Analysis of drug resistance of patients with tuberculosis after implementation of DOTS strategy in Hefei city. Modern preventive medicine 39:3951-3952,3955.

58. Tao BS (2012) Analysis on the sistuation of drug resistance of Mycobacterium tuberculosis in Beihai City. Journal of clinical and Experimental Medicine 11:966-967.

59. Chen XY,Mei SL,Lei LY,Tao Y,Wang XG,et al. (2013) Surveillance of drug resistance of tuberculosis in Lishui,Zhejiang. Disease surveillance 28:591-593.
