# Supplementary material for: The Prevalence of Drug-Resistant Tuberculosis in Mainland China: An Updated Systematic Review and Meta-Analysis
Source: PLoS One. 2016 Feb 9;11(2):e0148041. doi: 10.1371/journal.pone.0148041 (PMC4747587; doi:10.1371/journal.pone.0148041)
Supplement: S4 Table — (DOCX) [file pone.0148041.s008.docx]

**Table S4. Distribution of different TB drug resistance patterns among new and retreatment cases in China**

|  | **Mono-drug resistance /n**  **(95% CI)** | | | | **Multi-drug resistance/n**  **(95% CI)** | | | | **Resistance to specific drug * /n**  **(95% CI)** | | | |
| --- | --- | --- | --- | --- | --- | --- | --- | --- | --- | --- | --- | --- |
|  | **H** | **R** | **S** | **E** | **HR** | **HRS** | **HRE** | **HRSE** | **H** | **R** | **S** | **E** |
| **New cases** | 3.6/30  （3.0-4.4） | 1.2/30  （0.9-1.6） | 4.8/30  （3.8-6.1） | 0.6/30  （0.4-0.9） | 1.4/32（1.0-1.8） | 1.2/32  （0.9-1.6） | 0.7/32  （0.5-0.9） | 1.4/32  （1.1-1.9） | 12.0/48（10.6-13.6） | 6.6/48  （5.3-8.3） | 11.8/46  （10.1-13.7） | 3.7/46  （3.0-4.6） |
| **Retreatment cases** | 5.4/31  （4.3-6.8） | 3.1/31  （2.6-3.6） | 3.4/31  （2.5-4.8） | 1.4/31  （1.0-2.0） | 6.9/32  （5.2-9.0） | 5.6/32  （4.3-7.3） | 3.0/32  （2.2-4.2） | 8.5/32  （6.5-11.1） | 40.0/46（35.8-44.3） | 33.3/46  （29.6-37.2） | 28.4/44  (24.8-32.4) | 16.9/44  (13.8-20.5) |

Abbreviation: E, ethambutol; H, isoniazid; R, rifampicin; S, streptomycin.

* Resistance to specific drug regardless of mono-drug resistance or multi-drug resistance.
